# Dopamine and Somatostatin Analogues Resistance of Pituitary Tumors: Focus on Cytoskeleton Involvement

**DOI:** 10.3389/fendo.2015.00187

**Published:** 2015-12-22

**Authors:** Erika Peverelli, Donatella Treppiedi, Elena Giardino, Eleonora Vitali, Andrea G. Lania, Giovanna Mantovani

**Affiliations:** ^1^Endocrinology and Diabetology Unit, Department of Clinical Sciences and Community Health, Fondazione IRCCS Ca’ Granda Ospedale Maggiore Policlinico, University of Milan, Milan, Italy; ^2^Laboratory of Cellular and Molecular Endocrinology, IRCCS Clinical and Research Institute Humanitas, Milan, Italy; ^3^Endocrine Unit, IRCCS Humanitas Clinical Institute, University of Milan, Milan, Italy

**Keywords:** pituitary tumors, dopamine, somatostatin, DRD2, SSTRs, filamin A

## Abstract

Pituitary tumors, that origin from excessive proliferation of a specific subtype of pituitary cell, are mostly benign tumors, but may cause significant morbidity in affected patients, including visual and neurologic manifestations from mass-effect, or endocrine syndromes caused by hormone hypersecretion. Dopamine (DA) receptor DRD2 and somatostatin (SS) receptors (SSTRs) represent the main targets of pharmacological treatment of pituitary tumors since they mediate inhibitory effects on both hormone secretion and cell proliferation, and their expression is retained by most of these tumors. Although long-acting DA and SS analogs are currently used in the treatment of prolactin (PRL)- and growth hormone (GH)-secreting pituitary tumors, respectively, clinical practice indicates a great variability in the frequency and entity of favorable responses. The molecular basis of the pharmacological resistance are still poorly understood, and several potential molecular mechanisms have been proposed, including defective expression or genetic alterations of DRD2 and SSTRs, or an impaired signal transduction. Recently, a role for cytoskeleton protein filamin A (FLNA) in DRD2 and SSTRs receptors expression and signaling in PRL- and GH-secreting tumors, respectively, has been demonstrated, first revealing a link between FLNA expression and responsiveness of pituitary tumors to pharmacological therapy. This review provides an overview of the known molecular events involved in SS and DA resistance, focusing on the role played by FLNA.

## Introduction

Pituitary tumors are generally benign tumors that represent 10–25% of all intracranial neoplasms, and may cause visual field deficits and neurologic manifestations from mass effect, and/or endocrine syndromes caused by excessive pituitary hormone secretion, signs and symptoms depending from specific pituitary cell subtype origin of the adenoma. Prolactin (PRL)-secreting tumors are the most common of all functional pituitary tumors and cause amenorrhea, infertility, and galactorrhea in females, and impotence or infertility in males. Excessive secretion of growth hormone (GH) by tumorigenic somatotroph cells is the cause of gigantism during childhood and acromegaly in adults, with significant morbidity due to clinical complications involving cardiovascular, respiratory, and metabolic systems. Adenocorticotroph hormone (ACTH)-secreting tumors cause Cushing’s disease (hypercortisolism), and TSH-secreting tumors present with signs and symptoms of hyperthyroidism. Non-functioning pituitary tumors (NFPAs) are hormonally inactive, and patients with this tumor type often present with neurological symptoms due to the mass effect.

Pituitary tumors frequently preserve responsiveness to hypophysiotropic factors, including dopamine (DA) and somatostatin (SS), ubiquitous peptides that physiologically inhibit hormone secretion and cell proliferation at both the pituitary and the periphery levels, and thus are considered as molecules with therapeutical potential (1).

**Figure 1:**
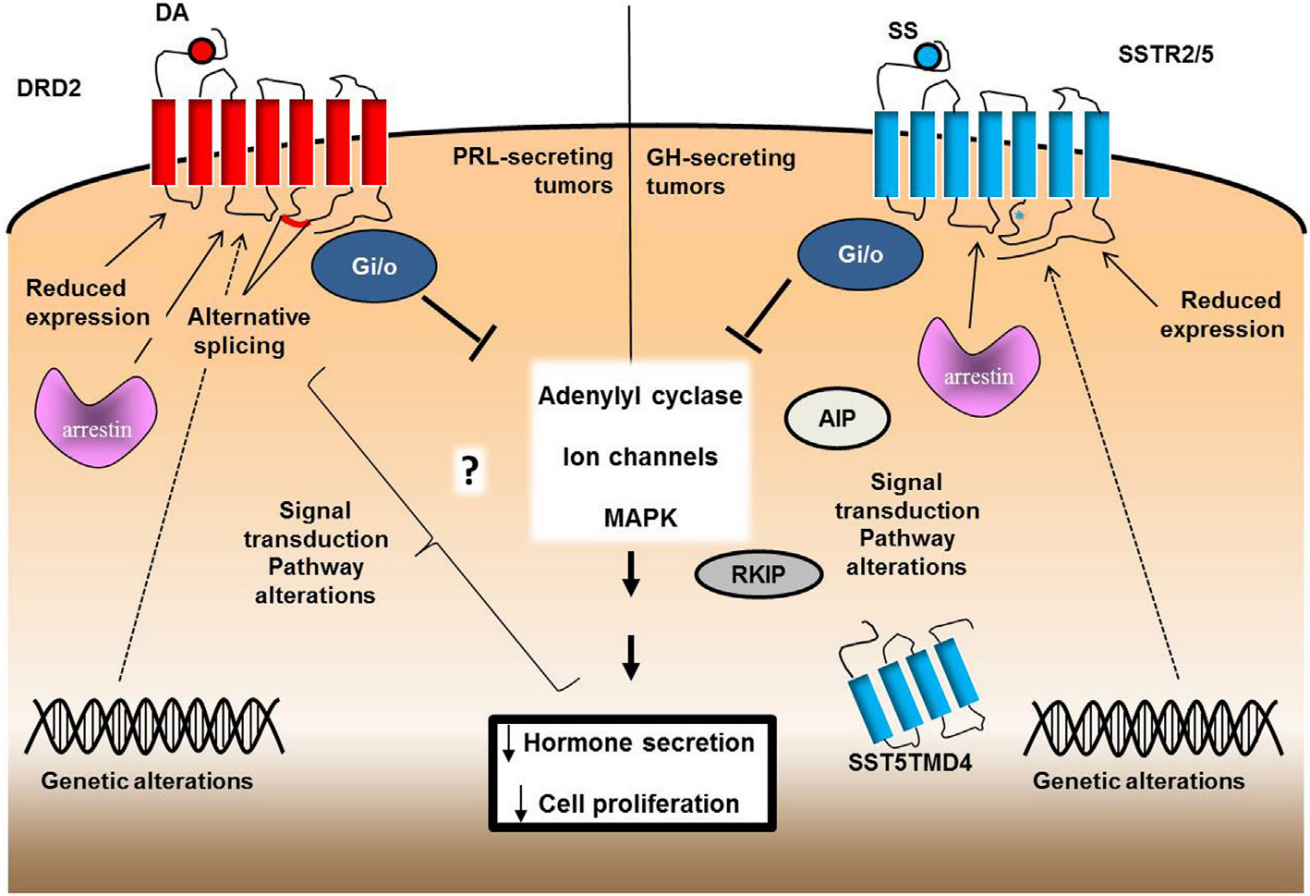
**Schematic representation of the possible molecular mechanisms involved in drug resistance of pituitary tumors**. DA-resistance in PRL-secreting tumors might be related to a defective expression of DRD2 or to an altered expression of specific splice variants. Genetic alterations of DRD2 or possible alterations in the molecules involved in receptor internalization or signal transduction culminating in the inhibitory action on PRL secretion and cell proliferation might also be involved. SS-resistance of GH-secreting tumors, besides to reduced SSTR2 and/or SSTR5 expression, rarely associated with mutations of SSTR2 and SSTR5 genes, but it has been correlated with the expression of truncated variatns of SSTR5 (SST5TMD4). Asterisk indicates the only mutational change found in the SSTR5 gene (R240W, in the third intracellular loop). Alterations in signal transduction may include G proteins, AIP, or arrestins.

The inhibitory actions of DA in pituitary tumors are mediated by DA receptor subtype 2 (DRD2) [reviewed in Ref. (2)] that inhibits both synthesis and secretion of PRL, by coupling with inhibitory heterotrimeric G proteins, G_i_ and G_o_, that in turn inhibit adenylyl cyclase and calcium channels. Moreover, DRD2 exerts antiproliferative effects through regulation of ERK1/2 activity (Figure 1). An alternative splicing generates two isoforms of this receptor, D2S (short) and D2L (long), possessing additional 29 residues in the third intracellular loop. DRD2 agonists, among which cabergoline is the most effective and best tolerated, are first-line therapy for prolactinomas as they are effective in controlling clinical symptoms, PRL levels, and tumor volume (3) and are used to a lesser extent in the treatment of ACTH-secreting tumors (4) and NFPAs (5).

Somatostatin binds to a family of G-proteins coupled receptors (SSTR1-5), among which subtypes 2 and 5 are expressed at high density in most GH-secreting pituitary tumors (6, 7). By coupling with multiple PTX-sensitive G proteins, SSTRs inhibit adenylyl cyclase activity and some subtypes reduce calcium entry by modulating L-type Ca^2+^ and K^+^ channels, all these events being involved in the reduction of hormone secretion [reviewed in Ref. (8)] (Figure 1). Both SSTR2 and SSTR5 mediate the antiproliferative effects of SS by tyrosine phosphatase activation and ERK1/2 phosphorylation inhibition, respectively, whereas only SSTR2 and SST3 subtypes mediate apoptotic effects (9–12). SS analogs, such as long-acting octreotide and lanreotide, are currently used in the treatment of pituitary tumors, particularly GH- and TSH-secreting tumors (13, 14).

## Drug Resistance of Pituitary Tumors: Molecular Mechanisms

Despite the demonstrated inhibitory effects on cell proliferation and hormone secretion of DA and SS analogs, a subset of patients (10% of patients with prolactinoma and 30% of acromegalic patients) are resistant to cabergoline and octreotide, respectively, while the large majority of patients with NFPA and ACTH-secreting pituitary tumors are unresponsive to both drugs (15–17).

Pharmacological resistance of PRL-secreting tumors is generally defined as failure to normalize PRL levels, and to achieve tumor size reduction of at least 50%, although this definition is still controversial and a variety of definitions of DA-resistance have been used (15, 18).

As far as acromegalic patients were concerned, definition of pharmacological resistance to SS analogs includes both biochemical remission (normal IGF1, random GH <1 μg/l, and a nadir GH after 75-g oral glucose tolerance test <0.4 μg/l) and tumor shrinkage (<20% of baseline volume) (17, 19).

Drug resistance of pituitary tumors might play a major impact on mortality, morbility, and quality of life. Moreover, prolactinomas resistance to cabergoline has been associated with invasive and more aggressive tumors, and with the increased risk to develop a carcinoma (15, 20).

Intensive investigations in the past years have discovered different molecular alterations possibly involved in this variable responsiveness to DA and SS, including defective expression or genetic alterations of DRD2 and SSTRs, alterations in their signaling pathways, and cytoskeleton proteins involvement (Figure 1) but much remains to be clarified about this complex phenomenon.

### Reduction of SSTRs and DRD2 Expression in Resistant Tumors

Generally, a reduced expression of functional DA and SS receptors in pituitary tumors has been associated with a poor response.

In particular, this event has been documented in prolactinomas, where a reduced density of DRD2 has been found in DA-resistant tumors vs. DA-sensitive tumors (21, 22). Lower expression of a specific isoform of DRD2, D2S (23, 24), or D2L (25) has also been documented in resistant vs. sensitive tumors.

More controversial is the association of SS resistance with the reduction of SSTRs expression, since data from literature suggest on the one hand that some GH-secreting tumors are resistant to therapy despite high SSTR2 expression, and on the other hand that the *in vivo* GH suppression and tumor shrinkage induced by SS analogs correlates with SSTR2 mRNA and protein levels (26–34).

Anyway, the molecular events responsible for reduced DRD2 and SSTRs expression are still largely unknown. Both genetic alterations and posttranslational mechanisms have been examined, and recently a key role for cytoskeleton proteins-mediated regulation has emerged, as discussed below.

### SSTRs and DRD2 Genetic Alterations

Published studies searching for genetic alterations of DRD2 and SSTRs have demonstrated that mutations in their coding sequence and/or loss of heterozigosity (LOH) in loci where they are located are very rare events.

In particular, no mutation of the DRD2 gene has been found in 79 prolactinomas (35), and a recently whole-exome sequencing on bromocriptine-resistant and -responsive prolactinomas demonstrated no sequence variants in DRD2 gene (36, 37). By genotyping about 200 patients with prolactinomas and controls for common DRD2 polymorphisms, DA-resistance has been correlated only to a common polymorphism, a synonymous cytosine to thymine transition at position 957, a change that has been associated with susceptibility to migraine and schizophrenia, and that might alter the predicted mRNA folding, leading to a decrease in mRNA stability and translation (38).

Similarly to DRD2, no mutations of SSTR2 and SSTR5 genes are usually found in resistant patients (39, 40). In fact, the only mutational change so far reported involving a SSTR is the germline R240W mutation in the SSTR5 gene, that was found in one acromegalic patient resistant to octreotide (41).

This missense mutation is located in the third intracellular loop of SST5, a critical region for G protein coupling, and functional studies have demonstrated that mutant R240W receptor failed to mediate the inhibition of GH release and cell proliferation induced by SSTR5 agonist, although it retained the ability to inhibit intracellular cAMP levels, as the result of a loss of coupling with G_oA_ protein but not with the other G proteins activated by wild type SSTR5, e.g., G_i1, i2, i3_ and G_oB_ (11, 42).

Polymorphic variants in SSTR2 gene seem to have no role in determining SS resistance of GH-secreting tumors. Two single nucleotide substitutions detected in the SSTR2 promoter sequence at positions −83 and −57 from the major transcription initiation site were not associated with differences in clinical characteristics, hormonal profile, or responsiveness to SS (43). Regarding SSTR5 gene, it has been found that T allele of rs34037914 single nucleotide polymorphism predisposes to resistance to antiproliferative effects of SS, increased aggressiveness, and post-surgical reoccurrence of pituitary tumors (44). However, LOH at the SSTR5 gene locus, located at chromosome 16p13.3, is a rare phenomenon, occurring in about 10% of pituitary tumors (45) and no mutational change in the retained allele was found in the GH-secreting tumor with LOH at the SSTR5 locus, LOH resulting associated with a normal responsiveness to octreotide (45).

Finally, the expression of a truncated variant of SSTR5 (SST5TMD4) that shows a preferential intracellular localization but is able to mediate signal transduction (46) has been found to correlate with a reduced ability of octreotide at normalizing hormone secretion in poorly responsive tumors *in vivo* (47) and with aggressive features, including SS-resistance and tumor invasiveness (48, 49). High levels of SST5TMD4 are correlated with poor responsiveness to SS analogs even in the presence of a high expression of SSTR2 (49), suggesting a dominant-negative effect of this truncated isoform of SSTR5 on SSTR2-mediated signaling.

### Post-Receptor Alterations

Beside alterations in SS and DA receptors, defects in the downstream signaling pathways activated by these receptors might be involved in the pharmacological resistance of pituitary tumors. The hypothesis of post-receptor alterations involved in resistance to SS analogs is supported by the dissociation between the antisecretory and antiproliferative effects of SS analogs observed *in vivo* in some acromegalic patients and in *in vitro* experiments in tumoral somatotroph cells where SS analogs exert antiproliferative effects without inhibition of GH secretion (50–52). Since SSTRs couple to different members of the inhibitory G proteins family to inhibit hormone secretion and cells proliferation, it is possible to hypothesize that alteration in the coupling with a specific G protein, or in a specific downstream effector, could determine the lack of activation of a specific pathway without affecting the other signaling cascades. In this regard, it has been shown that SSTR5 exerts antiproliferative and antisecretory effects in somatotroph by coupling with the inhibitory G protein G_oA_, independently of cAMP reduction, suggesting a dissociation between these pathways (11, 42).

Few data are available about a possible role of G proteins alterations in pituitary tumor resistance to pharmacologic treatment with DA or SS analogs. A reduction in G_i2_ protein, but not G_o_ and G_s_, expression has been described in cabergoline-resistant with respect to sensitive prolactinomas (53). Although a low expression of G_i1–3_ proteins has been reported in GH-secreting tumors (54), a possible correlation with SS responsiveness has not been analyzed. The alpha subunit of stimulatory G proteins deserves particular attention, since somatic activating mutations in its gene (GNAS, also called gsp oncogene) are found in 30–40% of GH-secreting pituitary tumors. It is worth noting that gsp-positive tumors are characterized by an increased responsivity to treatment with SS analogs, a feature up to now unexplained since no increase of SS receptors have been found in these tumors (40, 55–57).

Alterations in SSTRs signal transduction might be the cause of typical octreotide-resistance observed in acromegalic patients bearing germline mutations in aryl hydrocarbon receptor interacting protein (AIP) gene (58) or expressing low levels of AIP, even in the absence of mutations. AIP is a tumor-suppressor gene, which has been shown to induce tumor shrinkage via ZAC1 (59). Moreover, it has been shown that AIP inactivation leads to pituitary tumorigenesis through defective G_i_ signaling (60). It is of interest to note that low AIP expression does not associate with resistance to pasireotide (61), the SS analog with the highest affinity for SSTR5, suggesting that AIP is not involved in the signal transduction of this specific SSTR subtype.

Genetic analysis of AIP in 50 non-familial acromegalic patients resistant to treatment with SS analogs revealed a low, but non-negligible, prevalence of AIP germline mutations (62), suggesting a role for AIP alterations in SS-resistance of sporadic GH-secreting adenomas.

Fougner et al. reported an association of Raf kinase inhibitory protein (RKIP) expression with responsiveness of GH-secreting tumors to SS analogs. RKIP regulates Raf1 kinase activity, and consequently MAPK signaling, a pathway involved in mediating the antiproliferative effects of SS, and furthermore inhibits G protein receptor kinase 2, possibly affecting SSTRs internalization and degradation (63).

Beside activation of coupled G proteins, GPCRs are known to directly interact through their intracellular loops with cytoplasmic and surface proteins involved in GPCRs stabilization, desensitization, internalization, and signal transduction. Among these proteins, beta-arrestin 1 and 2 are scaffold proteins involved both in desensitization and signal transduction of several GPCRs, including SSTRs and DRD2 (64–66). Low expression of β-arrestin 1, but not β-arrestin 2, in GH- and PRL-secreting pituitary tumors correlates with a reduced recycling rate of SSTR2 and better SS analogs response in terms of GH suppression, both *in vitro* and *in vivo* (67).

A lesser amount of data is available on possible DRD2 signal transduction alterations. TGF-β/Smad pathway, that may in part mediate the antiproliferative effects of DA, was found down-regulated in DA-resistant prolactinomas compared to normal human anterior pituitaries (68). A recent whole-exome sequencing analysis of six bromocriptine-resistant and six responsive prolactinomas, in addition to exclude genetic variants of DRD2 gene, revealed sequence variants associated with 10 genes selected as potentially involved in resistance, among which PRB3 (36) and PRDM2 (37) were lower in resistant prolactinomas than in the responsive tumors, but the mechanisms involved are unknown. Finally, recent studies have identified a specific miRNA expression profile associated with bromocriptine-resistant prolactinoma. Among the differentially expressed miRNAs, mir-93 directly affected p21 expression in MMQ cells by targeting the 3′-UTR (69).

## Novel Mechanism in Drug Resistance of Pituitary Tumors: A Role for Cytoskeleton

Recently, cytoskeleton has emerged as novel player implicated in the complex mechanisms of pharmacological resistance of pituitary tumors to DA and SS.

Cell cytoskeleton not only plays a fundamental role in cell morphology maintenance, cell migration and adhesion, cell division, and cytoplasmic organelles localization and movement but it also has a role in extracellular signal transduction and in regulation of the activity of several receptors. The three main structural components of the cytoskeleton are microtubules, intermediate filaments, and microfilaments that originate from the polymerization of different monomers. These polymers that are characterized by structural and functional differences undergo continual turnover and rearrangement and specifically bind different families of partner proteins. Microfilaments, the thinnest of these filaments, are involved in cell morphology and motility and consist of actin filaments (F-actin) that are polymerized from monomeric globular actin (G-actin). Many families of actin-binding proteins that share an actin binding domain are involved in the regulation of polymerization/depolymerization processes and in crosslinking F-actin in bundles and networks. Among these, filamins (FLN) are actin crosslinking proteins that not only form orthogonal networks of F-actin but are also scaffold proteins able to bind different partner proteins.

### Filamin A Structure and Functions

The family of filamins consists of three homologous high-molecular weight proteins, filamin A, B, and C (FLNA, FLNB, and FLNC) that are encoded by different genes located on chromosome X, 3 and 7, respectively. These three isoforms of FLN in mammals show a strong homology in the entire coding sequences and possess highly conserved genomic organization (70).

Specifically, human FLNA, mapping to Xq28, is the first actin filament cross-linking protein identified in non-muscle cells (71) and is the most abundant filamin isoform in adults. FLNA as well as FLNB are ubiquitously expressed, contrary to FLNC, whose expression is restricted to skeletal and cardiac muscle. Several studies suggest that FLNs are essential for normal human development. Indeed, mutations in the respective genes cause a wide range of developmental malformations of the brain, bone, and heart with moderate to lethal consequences (72). In particular, FLNA mutations cause periventricular nodular heterotopia, a brain malformation due to abnormal neuronal migration, in which a subset of neurons fails to migrate into the developing cerebral cortex (73) or a wide spectrum of congenital malformations, such as otopalotodigital syndrome, frontometaphyseal dysplasia, and Melnick Needles syndrome (74).

The main role of FLNA, deriving from its ability to bind actin and omodimerize, is to stabilize F-actin network in a three-dimensional structure, but its functions are not limited to a structural role. Indeed, FLNA can bind many different partner proteins, including transmembrane proteins, such as several GPCRs, integrins, and ion channels, allowing their anchorage to actin cytoskeleton (75), but also intracellular signaling molecules, kinases, and transcription factors [revised in Ref. (76)], playing as important scaffold for signal transduction.

All these interactions are regulated by FLNA phosphorylation, proteolysis, mechanical forces, competition, and multimerization of partners.

**Figure 2:**
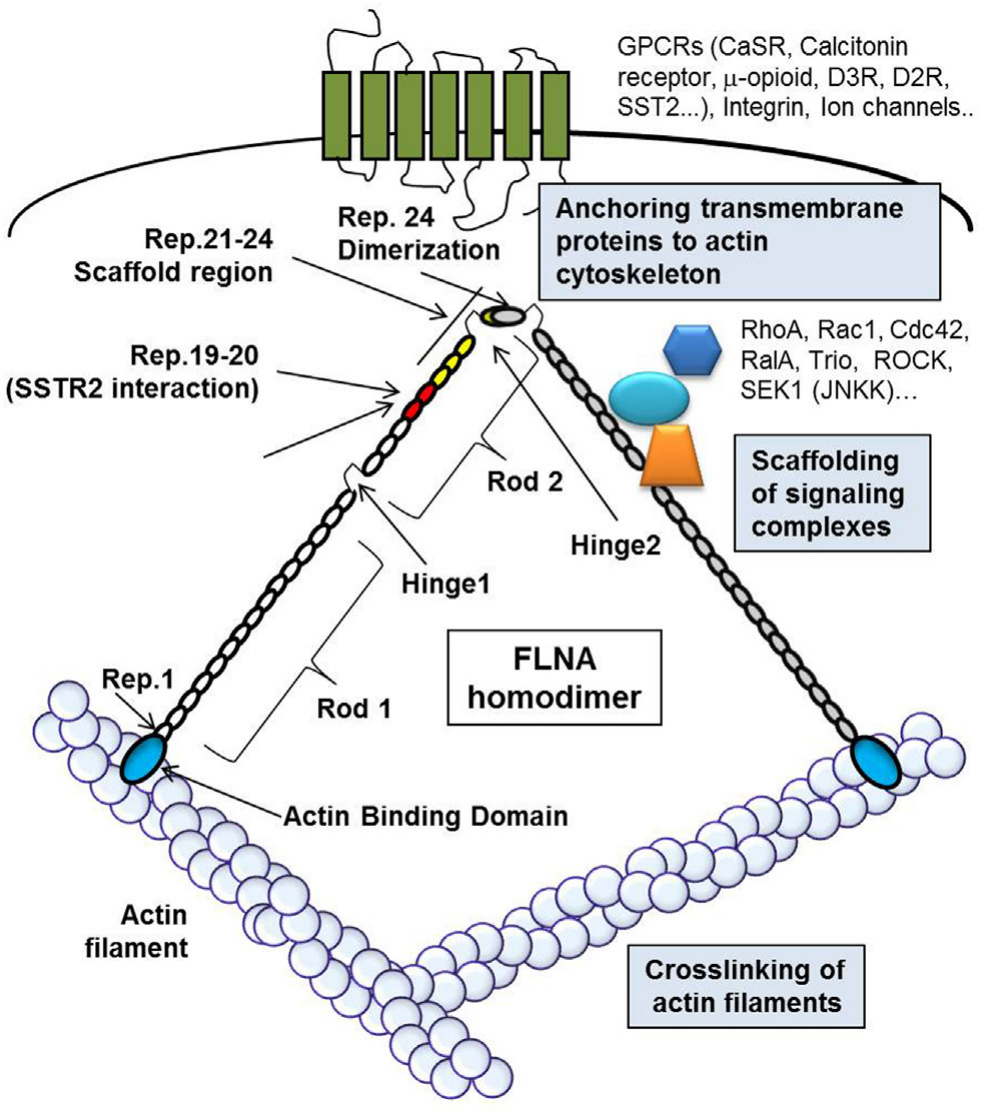
**The figure schematically represents FLNA structure and cellular functions**. The actin-binding domain (ABD, blue circle) is located at the N-terminus of each monomer of FLNA. The circles represent the Ig-like repeats of FLNA, in particular red circles represent repeats 19–20, involved in the interaction with DRD2 and SSTR2, and yellow circles represent the repeats 21–24, known as scaffold region for signal transduction molecules. The repeat 24 is the self-association domain that allows homodimer formation. FLNA crosslinks actin filaments, binds different transmembrane proteins, anchoring them to the actin cytoskeleton, and interacts with a variety of proteins involved in signal transduction.

These multiple functions of FLNA derive from its unique structure, schematically represented in Figure 2. FLNA is composed of two subunits of 280 kDa each that self-assemble, each monomer possesses an actin-binding domain (ABD) at the N-terminus, that consists of two calponin homology domains, followed by 24 immunoglobulin (Ig)-like repeats of about 96 amino acid residues. Two calpain-sensitive hinge regions (H1 and H2) separate the 24 repeats in a rod-1 domain (repeats 1–15), rod-2 domain (repeats 16–23), and repeat 24. A secondary ABD of lower affinity is located in the rod-1 domain, whereas rod-2 is involved in the interaction with partner proteins. The repeat 24 is the self-association domain that mediates FLNA homodimerization, allowing the formation of V-shaped flexible structures that results in the perpendicular cross-linking of actin filaments.

The diversity of the FLN family is increased by alternative splicing of FLN mRNA. Alternative poly(A) signals are employed and in some variants, there are internal deletions in some of the domains. In particular, previous studies have reported and alternative splicing of sequences encoding a region of eight amino acids in repeat 15 of FLNA, and an internal deletion of 41 amino acids between repeats 19 and 20 (residues 2127–2167) that identify FLNA variant-1 (77) that is widely expressed at low levels (70).

### FLNA Involvement in PRL-Secreting Pituitary Tumors Resistance to DRD2 Agonists

The interaction of FLNA with DRD2 was first demonstrated by Li and colleagues by a yeast two-hybrid screen and protein binding assays (78). Interestingly, this binding increased coupling efficiency of DRD2 to adenylate cyclase and plays a role in cell surface receptor clustering. Almost simultaneously, another study using the third intracellular loop of the DRD2 as bait in a yeast two-hybrid approach to screen a human brain cDNA library, confirmed a specific DRD2/FLNA association, better defining the specific regions of FLNA (repeat 19) and DRD2 (aminoacids 211–241 in the N-terminal region of the third intracellular loop) involved (79). FLNA and DRD2 colocalized in cell cultures of rat striatum. The authors found that DRD2 was predominantly intracellular in M_2_ cells, a human melanoma cell line does not express FLNA, whereas it localized at the plasma membrane in A_7_ cells, the same line stably transfected with FLNA, suggesting that FLNA is required for the cell surface localization of DRD2, as also demonstrated by using a dominant negative truncated form of FLNA (repeats 18–19, containing the DRD2, but not the actin, binding domain) (80).

**Figure 3:**
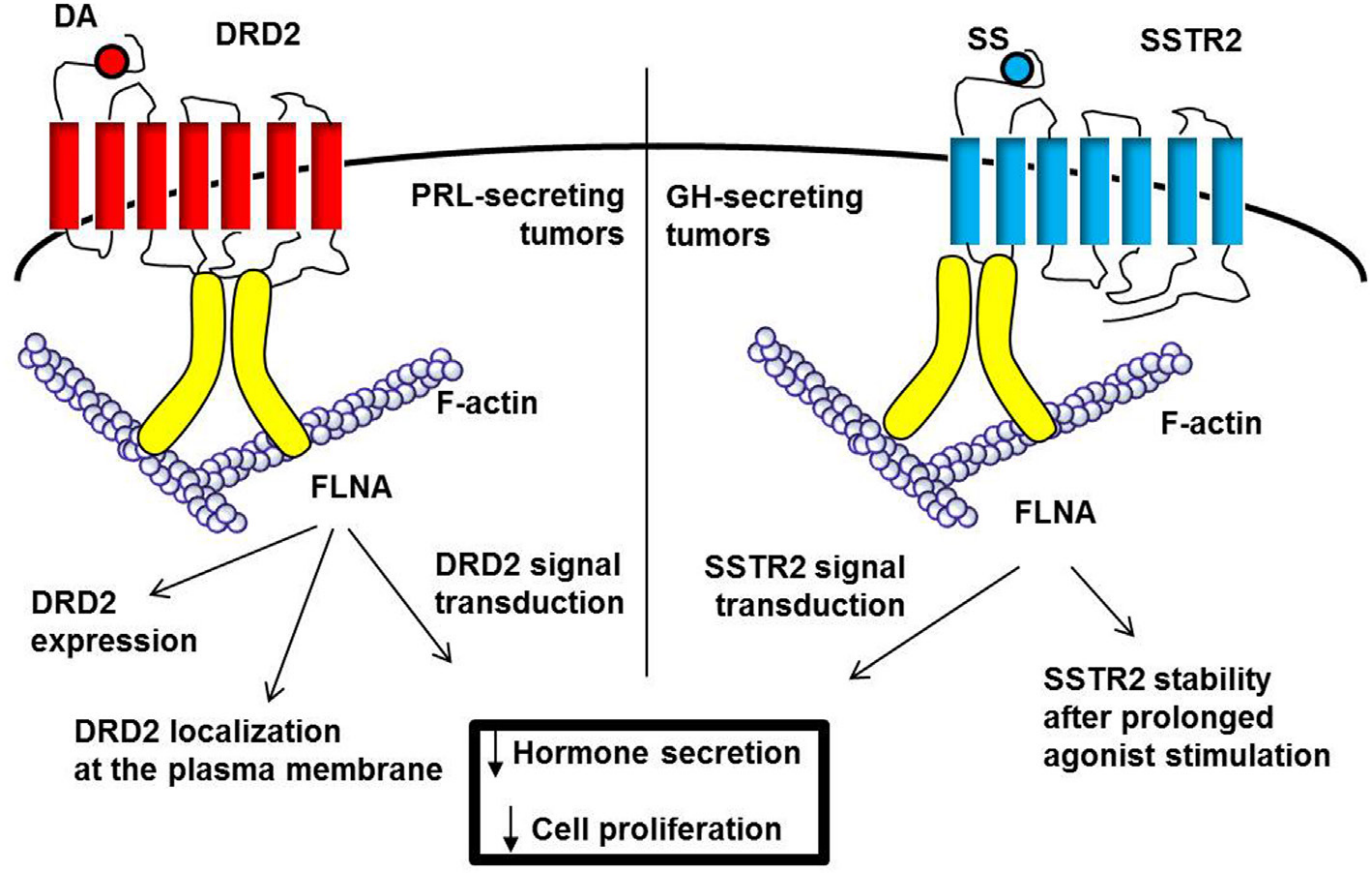
**FLNA is required for DRD2 and SSTR2 expression and signal transduction**. FLNA, schematically represented in yellow, bind DRD2 (third intracellular loop) and SSTR2 (first intracellular loop). In PRL-secreting tumors (left), FLNA binding is crucial for the correct localization at plasma membrane and expression of DRD2. In GH-secreting tumors (right), SSTR2 does not require FLNA for membrane localization, but in the absence of FLNA, SSTR2 expression is downregulated after prolonged agonist stimulation. Moreover, FLNA is required for both DRD2 and SSTR2 intracellular signaling leading to inhibitory effects on hormone secretion and cell proliferation.

Similar effects of FLNA on DRD2 regulation were also found in PRL-secreting pituitary tumors (Figure 3). First, it has been shown that in DA-resistant tumors the reduction of DRD2, previously associated with resistance, is accompanied by a low level of FLNA (81). *In vitro* experiments starting from this experimental observation demonstrated that alterations of FLNA levels in primary cultured prolactinoma cells by gene silencing or overexpression resulted in corresponding modifications of DRD2 levels, demonstrating that these two events are causally related. Furthermore, reduction of PRL release and ERK1/2 phosphorylation mediated by DRD2 agonist were impaired after FLNA silencing, whereas DA-resistant prolactinomas lacking FLNA recovered responsiveness when transfected with FLNA (81).

Experiments in rat MMQ cells, a cell model of prolactinoma endogenously expressing functional DRD2 and FLNA show that FLNA is not only required for DRD2 targeting to the cell membrane but also protects DRD2 against lysosomal degradation, suggesting a role for FLNA in the control of DRD2 fate toward recycling processes or lysosomal degradation (81). Since FLNA directly interacts with beta arrestins (82) that are involved in DRD2 trafficking (66), it is possible to hypothesize the formation of a complex receptor-FLNA–arrestin involved in the regulation of DRD2 stability. These data demonstrated a protective effect of FLNA from receptor instability, as also demonstrated for other receptors, including calcium-sensing receptor, calcitonin receptor, cystic fibrosis transmembrane conductance regulator, and the high-affinity IgG receptor FcgammaRI (83–86).

Beside a structural role in anchoring DRD2 to actin cytoskeleton and regulating receptor localization and stability, FLNA plays an additional functional role as scaffold for signaling molecules involved in DRD2 signal transduction. Indeed, since the pituitary has a substantial DRD2 reserve for PRL inhibition, and PRL response reaches the plateau at about 40% receptor occupancy in rat pituitary cells (87), the 60% reduction of DRD2 levels measured in prolactinomas and MMQ cells after FLNA knockdown might not entirely account for the loss of D2R effects on PRL release and cell proliferation (81).

Since FLNA is crucial for both DRD2 expression and signaling in lactotrophs, the loss of FLNA expression may be one of the mechanisms involved in loss of DA responsiveness previously documented in human prolactinomas.

Further studies are required to clarify the molecular events underlying FLNA reduced expression in resistant tumors. To date, no alterations in the FLNA gene CpG island with the highest probability to have regulatory functions have been found, excluding an epigenetic silencing (81).

### FLNA Regulates SSTR2 Expression and Signaling in GH-Secreting Pituitary Tumors

In addition to DRD2, FLNA seems to have a critical role also in the regulation of SSTR2, with important implications for SS responsiveness in acromegalic patients.

A direct interaction of FLNA with SSTR2 has been first demonstrated by surface plasmon resonance (88). Specifically, this interaction involve SSTR2 first intracellular loop and FLNA repeats 19–20 and plays a role in SSTR2 stabilization at the cell membrane. Contrary to what observed for DRD2, SSTR2 was correctly targeted to the plasma membrane in M_2_ cells lacking FLNA, but after agonist stimulation SSTR2 internalization rate in M_2_ cells is increased with respect to A_7_ cells. The authors also found that FLNA is required for SSTR2 mediated inhibition of cell survival, and the molecular mechanism involves a competition of FLNA with p85, the regulatory subunit of PI3K, for direct binding to SSTR2. Ligand-stimulated FLNA binding results in the disruption of the SSTR2-p85 complex and the subsequent inhibition of PI3K (88).

The importance of FLNA in SSTR2 regulation in GH-secreting pituitary tumors has recently been examined (89) (Figure 3). In contrast to DRD2 in prolactinomas, the expression of FLNA in GH-secreting tumors did not correlate with SSTR2 levels, and FLNA silencing in human tumoral cells did not affect SSTR2 expression and membrane localization. However, by using different FLNA dominant negative mutants that selectively abolished the ability of FLNA to interact with SST2 (FLNA repeats 19–20) or to function as scaffold for partner proteins (FLNA 21–24), the authors demonstrated that FLNA plays both a structural role, by stabilizing SST2 expression after prolonged stimulation with a specific agonist, and a functional role, acting as scaffold protein for molecules involved in signal transduction.

Using as a model pituitary GH-secreting rat GH3 cells, the authors showed that disrupting the FLNA/SSTR2 interaction, receptor stability after prolonged agonist stimulation was strongly compromised. The mechanism probably involve an effect of FLNA in regulating lysosomal degradation of SSTR2, since SST2 ligand-induced down-regulation was abolished when lysosomal degradation was prevented. An effect of FLNA in regulating SSTR2 internalization rate was also reported (88).

Furthermore, *in vitro* experiments in tumoral somatotrophs demonstrated that FLNA is required for SSTR2-induced reduction of cyclin D1 and caspase3/7 activation, suggesting a crucial role for FLNA in mediating antiproliferative and proapoptotic effects of SSTR2. The observation that FLNA 21–24 which does not abolish the interaction of SSTR2 with endogenous FLNA, was able to block the apototic effects of SST2 and the SST2-mediated ERK1/2 inhibition suggests that the FLNA scaffold properties are required for the assembly of signal transduction complexes.

Overall, these data support a new role for FLNA in the responsiveness of patients with GH-secreting pituitary tumors to pharmacological treatment with SS analogs. Low levels of FLNA, causing loss of coupling of SSTR2 with downstream signal transduction molecules, might explain the resistance to SS analogs in GH-secreting pituitary tumors even if in the presence of SSTR2.

It is of interest to note that FLNA splice variant-1, that was found widely expressed at low levels, lacks 41 amino acids between repeats 19 and 20, the region involved in the interaction both with DRD2 and SST2. NMR spectroscopy analysis has revealed that in the splice variant the repeat 19 is intrinsically unfolded (90), with consequent biological effect. FLNA splice variant-1 showed an increased binding to integrins with respect to wild-type non-spliced FLNA, suggesting that this alternative splicing might function as a regulatory mechanism for the binding of partner proteins to FLNA, with possible implications in pituitary tumors responsiveness to SS/DA analogs.

### Possible Role of FLNA in Pituitary Tumor Aggressiveness and Invasiveness

Beside its role in GPCR regulation, FLNA is involved in the control of cell motility. Indeed, FLNA is recruited to membrane protrusions of migrating cells where it functions as scaffold for proteins involved in cell migration and adhesion.

The observation that in periventricular heterotopia FLNA null mutations caused impaired neuronal migration within the cerebral cortex (73), together with experimental evidences obtained in FLNA-deficient M_2_ cells that are not able to migrate (91), suggest that FLNA is crucial in promoting cell migration. In contrast, it has been shown that overexpression of FLNA inhibited cell motility (92) and FLNA knockdown promoted cell migration, invasion, and metastasis (93, 94), leading to the conclusion that FLNA positive or negative effect on cell motility depend on the cell type and on a balanced level of FLNA expression.

Pituitary tumors are generally benign, but frequently present local invasiveness that strongly reduces neurosurgery success. Since DA-resistance of prolactinomas has been associated with increased invasiveness and aggressiveness of these tumors (15, 20), and reduced FLNA levels correlate with resistance to dopaminergic drugs, it is possible to hypothesize that low levels of FLNA might be involved in pituitary tumors invasive behavior.

Up to now, no data are present in literature about the effects of FLNA expression levels on pituitary cell migration and invasion, and further studies are required to test a possible contribution of FLNA alterations in the invasiveness, aggressive behavior, and recurrences of pituitary tumors.

## Conclusion

Molecular mechanisms underlying the resistance of pituitary tumor to pharmacological treatment with DA and SS analogs might involve different steps that from the receptor activation by agonist lead to the final biological responses in terms of hormone secretion inhibition as well as tumor shrinkage. In the absence of genetic defect in SSTR2, SSTR5, and DRD2 genes, alterations have been reported in molecules involved in the intracellular signal transduction pathway specific of these receptors, and in the regulation of receptors expression. Growing evidence revealed that DRD2 and SSTR2 expression, localization, and signaling are regulated by interaction with cytoskeleton protein FLNA, once again emphasizing the multiple roles of cytoskeleton in physiological cell functions. Future works searching for genetic and post-translational modifications affecting FLNA expression and function, as well as for a possible involvement of other cytoskeleton proteins, might help to clarify the complex mechanisms involved in pituitary tumors pharmacological resistance.

## Author Contributions

EP: preparing the manuscript, manuscript revision, final approval of the version to be published. DT, EG, EV, AL: preparing the manuscript, final approval of the version to be published. GM: manuscript revision, final approval of the version to be published.

## Conflict of Interest Statement

The authors declare that the research was conducted in the absence of any commercial or financial relationships that could be construed as a potential conflict of interest.
